# Decellularized Human Chorion Membrane as a Novel Biomaterial for Tissue Regeneration

**DOI:** 10.3390/biom10091208

**Published:** 2020-08-20

**Authors:** Laura P. Frazão, Joana Vieira de Castro, Cristina Nogueira-Silva, Nuno M. Neves

**Affiliations:** 1 I3B’s—Research Institute on Biomaterials, Biodegradables and Biomimetics of University of Minho: 3Bs Research Group, 4805-017 Guimarães, Portugal; laurafrazao@i3bs.uminho.pt (L.P.F.); joana.castro@dep.uminho.pt (J.V.d.C.); 2 ICVS/3B’s—PT Government Associate Laboratory, Braga/Guimarães, Portugal; cristinasilva@med.uminho.pt; 3 Life and Health Sciences Research Institute, School of Medicine, University of Minho, 4710-057 Braga, Portugal; 4 Department of Obstetrics and Gynecology, Hospital de Braga, 4710-243 Braga, Portugal

**Keywords:** chorion membrane, decellularization, extracellular matrix, biocompatible, tissue regeneration

## Abstract

Although some placenta-derived products are already used for tissue regeneration, the human chorion membrane (HCM) alone has been poorly explored. In fact, just one study uses decellularized HCM (dHCM) with native tissue architecture (i.e., without extracellular matrix (ECM) suspension creation) as a substrate for cell differentiation. The aim of this work is to fully characterize the dHCM for the presence and distribution of cell nuclei, DNA and ECM components. Moreover, mechanical properties, in vitro biological performance and in vivo biocompatibility were also studied. Our results demonstrated that the HCM was successfully decellularized and the main ECM proteins were preserved. The dHCM has two different surfaces, the reticular layer side and the trophoblast side; and is biocompatible both in vitro and in vivo. Importantly, the in vivo experiments demonstrated that on day 28 the dHCM starts to be integrated by the host tissue. Altogether, these results support the hypothesis that dHCM may be used as a biomaterial for different tissue regeneration strategies, particularly when a membrane is needed to separate tissues, organs or other biologic compartments.

## 1. Introduction

The human placenta is usually considered a biological waste. Nevertheless, it is consistently available from full-term births and it is also a source of mesenchymal stem cells, growth factors and extracellular matrix (ECM) proteins [1,2].

Some placenta-derived products are already used for tissue regeneration [1,3,4,5,6,7,8,9,10,11,12,13]. Nevertheless, when non-decellularized, they are only used in poorly vascularized sites, such as some wounds, tendon, ligament and cartilage [13]. So, one way to obtain ECM proteins and avoid a host adverse immune response when implanted in vivo is through tissue decellularization [14,15,16,17,18]. In fact, the human placenta has been fully decellularized through the creation of ECM suspensions to further create membranes [19] or sponges [20]. In these works, the various components of the human placenta are not differentiated, such as the human amniotic membrane (HAM) and the human chorion membrane (HCM) [21]. Moreover, the native tissue architecture is not maintained. Nevertheless, some works maintained the native tissue architecture after decellularization, for the whole placenta [3] and for the HAM [4,5,6,8,9,10,22,23,24] and HCM alone [7].

Decellularized HAM alone is widely characterized and studied and has been shown to induce osteogenic differentiation of human dental apical papilla cells [4], to be a good chondrocyte substrate/carrier [22], and to promote epithelization [5,10,23]. Moreover, decellularized HAM was used in cell-matrix adhesion studies, to produce skin equivalents, and as a pericardial substitute [6,8,9,10,24]. On the other hand, HCM alone as a biomaterial for tissue regeneration is still largely unexplored. Although HCM has been used as a reservoir of stem [25] and proangiogenic [26] cells, and as a source of small-diameter vascular grafts [1], only one study uses decellularized HCM (dHCM) with native tissue architecture (i.e., without ECM suspension creation) as a substrate for cell differentiation [7]. However, in that study, the dHCM is poorly characterized for the presence of ECM components, mechanical properties, in vitro cytocompatibility and in vivo biocompatibility.

The purpose of this study is to fully characterize the dHCM for the presence and distribution of cell nuclei, DNA and ECM components. Finally, the mechanical properties, and the in vitro and in vivo biocompatibility of dHCM were characterized envisioning its use as an ECM-based scaffold able to guide tissue regeneration, particularly when a membrane is needed to separate tissues, organs or other biologic compartments.

## 2. Materials and Methods

### 2.1. Human Placentas

Human placentas used in this study were collected from elective and term cesarean sections performed in the Obstetrics Department of the Hospital de Braga. The study follows a protocol approved by the ethical committee of the Hospital de Braga and of the University of Minho (CESH 030/2016, SECVS 136/2015, respectively) and following all the international best practices for research with human biological samples. An informed consent was signed by all the donors. Sterile conditions were maintained during all processing steps. Placentas were stored in Dulbecco’s phosphate-buffered saline (D-PBS) with 10% antibiotic/antimycotic (#15240062, Thermo Fisher Scientific, Waltham, MA, USA) at 4 °C for a maximum of 1 day. HCM was separated from the amnion, washed with phosphate-buffered saline (PBS) to remove any blood and stored at −80 °C until further use.

### 2.2. Decellularization of Human Chorion Membrane (HCM)

As previously described by us [27], HCM were submitted to two freezing/thawing cycles (−80 °C and 37 °C, respectively) and, subsequently, were treated with different concentrations (0.5% and 0.1%) of sodium dodecyl sulfate (SDS) (#MB18101, NZYTech, Lisbon, PT) solution in ultra-pure water. HCM were submitted to three washes with 0.5% SDS for 2 h each. Then, the membranes were washed overnight with 0.1% SDS. Three washes of 15 min were performed with 1% Triton-X100 (#A16046, Thermo Fisher Scientific, Waltham, MA, USA) in PBS. All treatments were done at 4 °C in an orbital shaker at 110 rpm. The membranes were then scraped on both sides and the trophoblast layer (TL) was removed and further used for collagen and glycosaminoglycans (GAGs) quantification. In order to identify both membrane sides (trophoblast and reticular layer), the membranes were marked with a knot with suture line. Subsequently, membranes were treated with 0.001 mg/mL DNase I (#A3778 PanReac AppliChem ITW Reagents, Darmstadt, DE, Germany), for 30 min at 37 °C, followed by a 30 min wash with 0.1% SDS at 4 °C. Finally, the membranes were washed with PBS at least three times, for 2 h each, at 4 °C. The entire process was done under sterile conditions. After decellularization, the membranes were stored in PBS with 1–2% antibiotic/antimycotic at 4 °C.

### 2.3. DNA Extraction and Quantification

Membranes were air dried and weighed and total DNA from both native and decellularized HCM (dHCM) was extracted using the DNeasy Blood and Tissue Kit (#69504, Qiagen, Hilden, Germany), according to manufacturer’s instructions. The quantification of double-stranded DNA (dsDNA) was performed using the Quant-iT PicoGreen dsDNA Assay Kit (#P7589, Invitrogen, Carlsbad, CA, USA), according to manufacturer’s instructions. Electrophoresis was performed to assess the size of the DNA fragments, using a 1% agarose gel and GeneRuler DNA Ladder Mix (#SM0334, Thermo Fisher Scientific, Waltham, MA, USA). Four independent samples were used in each condition.

### 2.4. Histological Analysis

The samples were fixed in 10% neutral-buffered formalin at 4 °C (for at least 24 h), embedded in paraffin, and 5 μm thick transverse sections were cut with a microtome. Histochemical stains such as hematoxylin and eosin (H&E) and Masson’s trichrome were performed in the samples. For H&E staining, samples were stained with hematoxylin (#7212, Thermo Fisher Scientific, Waltham, MA, USA) for 1 min, washed for 30 s and stained with eosin for 10 min (#71204, Thermo Fisher Scientific, Waltham, MA, USA). For Masson’s trichrome staining, slides were submitted to Azure B solution for 5 min, stained with hematoxylin for 5 min and washed in picric ethanol for 5 min. After that, samples were stained in Biebrich Scarlet-Acid Fuchsin for 15 min, submitted to 1% phosphomolybdic acid for 5 min and to Aniline Blue for 4 min. After each staining, all slides were washed with water, let to dry and rinsed with alcohol, cleared in xylene and mounted in Entellan rapid (#107960, Merck, Darmstadt, DE, Germany). Slides were observed under an optical microscope with a coupled digital camera (DM750, Leica, Wetzlar, DE, Germany). At least three independent samples were used in each condition.

### 2.5. Scanning Electron Microscopy (SEM)

Samples were fixed with 2.5% glutaraldehyde in PBS. After three PBS washes, samples were dehydrated with increasing concentrations of ethanol (10% to 100%). Samples were air dried and mounted in SEM pins using carbon tape. The samples were coated with gold using a Sputter Coater (#EM ACE600, Leica, Wetzlar, DE, Germany). Micrographs were collected with a scanning electron microscope with EDS (#JSM-6010 LV, JEOL, Akishima, Tokyo, Japan). At least three independent samples were used in each condition.

### 2.6. Swelling Assay

Initially, dry samples were weighed and then were immersed in D-PBS or culture medium. At different time-points (0.5 h; 1.5 h; 2.5 h; 3.5 h; 4.5 h; 5.5 h), samples were weighed immediately after the excess of liquid was removed by putting them between two pieces of filter paper. Samples were reimmersed in liquid until the next time-point. The process was repeated until the equilibrium was reached (stable percentage of water uptake). Six samples were used in each condition.

### 2.7. Collagen Quantification

The collagen content of HCM, dHCM and trophoblast layer (TL) was extracted and quantified using the Sircol Collagen Assay Kit (#S5000, Biocolor, Carrickfergus, ATM, UK) for soluble collagen and the Sircol Insoluble Collagen Assay Kit (#2000, Bicolor, Carrickfergus, ATM, UK) as described before [28] and according to manufacturers’ instructions. Three independent samples were used in each condition.

### 2.8. Sulfated Glycosaminoglycans Quantification

Sulfated glycosaminoglycans (GAGs) content of HCM, dHCM and TL was quantified as previously described [29]. Samples were digested with 0.5 mg/mL papain (#P4762, Sigma-Aldrich, St. Louis, MO, USA) and the supernatant was stained with 1,9-dimethylmethylene blue (DMB). A dilution series of chondroitin sulfate in distilled water (50 μg/mL) was used as standard solution. Samples were diluted 1:10 in distilled water before measurement. A mixture of 20 μL standards and diluted samples and 250 μL of DMB was added to a 96-well plate, and absorbance was measured immediately at 525 nm using a microplate reader (Synergy HT, Biotek, Winooski, VT, USA). Three independent samples per condition were analyzed.

### 2.9. Protein Extraction and Quantification

Soluble protein was extracted from HCM and dHCM using Tissue Extraction Reagent I (#FNN0071, Thermo Fisher Scientific, Waltham, MA, USA) with a protease inhibitor cocktail (#P8340, Sigma-Aldrich, St. Louis, MO, USA). The soluble protein content was determined using Protein Assay Dye Reagent Concentrate (#5000006, Biorad, Hercules, CA, USA), according to manufacturer’s instructions.

### 2.10. SDS-PAGE

The SDS Gel Preparation Kit (#08091, Sigma-Aldrich, St. Louis, MO, USA) was used to prepare the 4% stacking gel and 9% running gel. For each sample, 0.5 μg/μL of protein was loaded in the respective well. Following SDS-PAGE, the gel was stained with Coomassie Blue R-250 (HS-604, National Diagnostics, Atlanta, GA, USA) and an image was obtained using a Transilluminator (Biospectrum AC Chemi HR 410, UVP). At least three independent samples were used in each condition.

### 2.11. Dot Blot

One drop of each sample of soluble protein was placed in a nitrocellulose membrane. After drying, membranes were incubated for 1 h with 5% BSA, with agitation, at room temperature. Subsequently, membranes were incubated with primary antibody:mouse anticollagen type I 1:1000 (#ab90395, abcam, Cambridge, UK); rabbit anticollagen type IV, 1:500 (#ab6311, abcam, Cambridge, UK); rabbit antifibronectin 1:500 (#ab45688, abcam, Cambridge, UK); and mouse antilaminin, 1:500 (#L8271, Sigma-Aldrich, St. Louis, MO, USA). After overnight incubation, membranes were washed 3× for 5 min with Tris Buffered Saline (TBS) with Tween 20 and then the R.T.U. VECTASTAIN^®^ Universal ABC Elite^®^ Kit (#PK-7200, Vector Laboratories, Burlingame, CA, USA) was used as a secondary antibody, in accordance with manufacturer’s instructions. Finally, incubation was revealed using a Peroxidase Substrate Kit (DAB) (#SK-4100, Vector Laboratories, Burlingame, CA, USA). Extraction buffer without samples was used as a negative control. Collagen type I (#sc-136157, Santa Cruz Biotechnology, Santa Cruz, CA, USA), collagen type IV (#C5533, Sigma-Aldrich), fibronectin (#FC010, Sigma-Aldrich, St. Louis, MO, USA) and laminin (#L6274, Sigma-Aldrich, St. Louis, MO, USA) were used as positive controls. At least three independent samples were used in each condition.

### 2.12. Immunolocalization of Proteins

Immunolocalization of different proteins, such as collagen type I, collagen type IV, fibronectin and laminin, was performed in paraffin-embedded samples sectioned at 5 μm, as previously described for collagen type II [30]. Briefly, samples were incubated with mouse anticollagen type I 1:100 (#ab90395, abcam, Cambridge, UK); rabbit anticollagen type IV, 1:50 (#ab6311, abcam, Cambridge, UK); rabbit antifibronectin 1:300 (#ab45688, abcam, Cambridge, UK); and mouse antilaminin, 1:300 (#L8271, Sigma-Aldrich, St. Louis, MO, USA), overnight at 4 °C in a humidified atmosphere. As a secondary antibody, the R.T.U. VECTASTAIN^®^ Universal ABC Elite^®^ Kit (#PK-7200, Vector Laboratories, Burlingame, CA, USA) was used, in accordance with manufacturer’s instructions. Incubation was revealed using a Peroxidase Substrate Kit (DAB) (#SK-4100, Vector Laboratories, Burlingame, CA, USA). Samples were counterstained with hematoxylin and mounted in an aqueous mounting medium. Slides were observed in an optical microscope with a coupled camera (DM750, Leica, Wetzlar, DE, Germany). At least three independent samples were used in each condition.

### 2.13. Mechanical Properties

Static mechanical properties were assessed using universal mechanical testing equipment (#5543, INSTRON, Norwood, MA, USA) equipped with a 1 kN load cell. Nine samples of HCM and dHCM were cut in pieces of 20 × 5 mm and mounted in specific cassettes (to prevent the clamping system from damaging the samples), and the specimens were hydrated. The strain rate was defined at 5 mm/min and a 10 mm gauge length was used in tensile tests. Tests were finished when the specimens were ruptured.

### 2.14. Cytotoxicity Analysis

Human umbilical vein endothelial cell line EA.hy926 and human lung fibroblast cell line MRC-5 were cultured in complete medium (Dublecco’s Modified Eagle’s Medium (DMEM) with 10% fetal bovine serum (FBS; #A3160801, Thermo Fisher Scientific, Waltham, MA, USA) and 1% penicillin/streptomycin (#15240062, Thermo Fisher Scientific, Waltham, MA, USA) in T150 flasks at 37 °C in a humidifier incubator with 5% CO_2_. After reaching 80% of confluence, the cells were seeded into inserts. dHCM were mounted in cell crown inserts for 24-well plates (#Z742380-12EA, Sigma-Aldrich, St. Louis, MO, USA). Millicell hanging cell culture inserts, Polyethylene terephthalate (PET) 0.4 µm for 24-well plates (#MCHT24H48, Millipore, Burlington, MA, USA), were used as control. Both inserts were immersed in culture medium overnight before cell seeding. EA.hy926 and MRC-5 cells were seeded on the inserts with a density of 20 × 10^3^ cells/cm^2^. Metabolic activity was evaluated by MTS assay (CellTiter 96 AQueous One Solution, Promega, Madison, WI, USA). Three independent assays for each cell line were performed.

### 2.15. In Vivo Studies

#### 2.15.1. Animals and Ethical Issues

The experiments were performed in male BALB/c mice aged 9 weeks old (Charles Rivers, Barcelona, Spain), and a total of 20 animals were used. Animals were housed five per cage in a limited-access rodent facility. Food and water were available ad libitum and animals were maintained in a climate-controlled room (22.0 ± 0.5 °C temperature) in 12/12 h light/dark cycle with lights on at 8:00 a.m. The experimental protocol was approved by the Institutional Ethical Commission and followed the European Community Council Directive concerning the use of animals for scientific purposes. Before surgery, animals were exposed to handling twice a day. All efforts were made to minimize animal suffering and to use only the number of animals necessary to produce reliable scientific data.

#### 2.15.2. Subcutaneous Implantation

The main goal of this in vivo study was the assessment of the biocompatibility of dHCM membranes in healthy animals. BALB/c mice were anesthetized by an intraperitoneal injection of Domitor (medetomidine 0.5 mg/kg) plus Imalgene (ketamine 75 mg/kg). The dorsum of the animals (implantation area) was shaved, cleaned and sterilized with Betadine^®^ and two skin incisions were made (each 0.5 cm length) in each mouse. A sample of the dHCM was implanted subcutaneously in each of the pockets and the incision was closed using 5-0 sutures (15 animals, 5 animals for each time-point). Empty defects were used as controls, either in the control group (5 animals, 2 animals for the first two time-points and 1 animal in the last time-point) and in the contralateral side of the pocket containing the material. After the procedure, the anesthesia was reversed with a subcutaneous injection of antisedan and the animals were monitored until fully awake (eating and grooming). After 4, 10 or 28 days, animals were euthanized by pentobarbital injection, and dHCM membranes and surrounding tissues were explanted for subsequent histological analyses.

### 2.16. Statistical Analysis

Statistical analysis was performed using GraphPad Prism 7. The Shapiro–Wilk test was used to assess data normality. When data followed a normal distribution, parametric tests were used, namely the unpaired t-test and two-way ANOVA test followed by Tukey’s multiple comparisons test. When data did not follow a normal distribution, the Mann–Whitney test was used. *p* < 0.05 was considered statistically significant.

## 3. Results

### 3.1. HCM Decellularization and ECM Protein Retention

**Figure 1:**
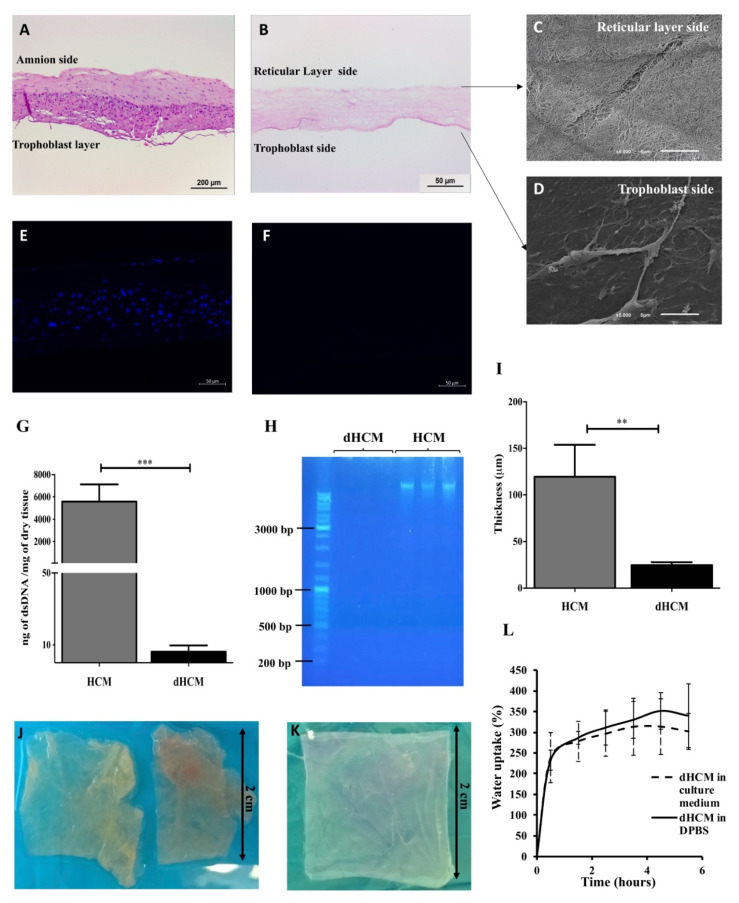
Human chorion membrane decellularization. Representative transversal sections of hematoxylin/eosin (H&E) staining of human chorion membrane (HCM) (**A**) and decellularized HCM (dHCM) (**B**). Representative scanning electron microscope (SEM) micrographs of dHCM from the reticular layer side (**C**) and the trophoblast side (**D**). Representative transversal sections of DAPI staining from HCM (**E**) and dHCM (**F**). Double-stranded DNA (dsDNA) quantification in HCM and dHCM (**G**). Agarose gel electrophoresis of DNA extracted from HCM and dHCM (**H**). Thickness of air-dried HCM and dHCM measured with a picometer in at least three different sites (**I**). Top view of HCM (**J**) and dHCM (**K**) cut into pieces of 2 × 2 cm. Swelling behavior of dHCM in culture medium and Dulbecco’s phosphate-buffered saline (D-PBS) (**L**). ** *p* ≤ 0.01 and *** *p* ≤ 0.001.

The efficiency of the decellularization protocol was assessed and the results are summarized in Figure 1. In the H&E staining, no nuclei can be observed in the dHCM when compared with native tissue (Figure 1A,B). Moreover, the chemical and physical processes used in the decellularization process completely removed the HCM’s trophoblast layer and the nuclei from the reticular layer (Figure 1A,B). SEM images revealed that the dHCM is composed by nanofibers in the reticular layer side (Figure 1C), however, a thin compact layer covered the dHCM in the trophoblast layer side of the membrane (Figure 1D). Cellular removal in the dHCM (Figure 1F) was also corroborated by 4′,6-Diamidino-2-Phenylindole (DAPI) staining in which no signal was detected compared with the native membrane (Figure 1E). Additionally, DNA quantification and length of DNA fragments showed a significant removal of DNA (*p* = 0.0002) in dHCM when compared with HCM (Figure 1G). The dHCM presented a DNA content around 10 ng of dsDNA/mg of dry tissue (Figure 1G). DNA content of dHCM was so low that no DNA was visible on the agarose gel in lanes loaded with dHCM, compared with the large amounts of DNA in lanes loaded with native tissue (Figure 1H). Thickness measurement of air-dried HCM and dHCM was also performed. After the decellularization protocol, the membrane became five times thinner (119.50 ± 34.32 μm of HCM compared to 24.50 ± 3.11 μm of dHCM) (*p* = 0.0150; Figure 1I). Although there were large differences in tissue thickness between native and decellularized tissue, a compact and easy to manipulate membrane was obtained (Figure 1J,K).

The swelling behavior of dHCM in culture medium and PBS is shown in Figure 1L. In the first 30 min, an increase of 230–240% of dHCM weight was observed. After this time, the swelling behavior stabilized around 300–350%. Moreover, when wet, dHCM thickness increased to 36.00 ± 8.03 μm (*p* = 0.0159; data not shown).

**Figure 2:**
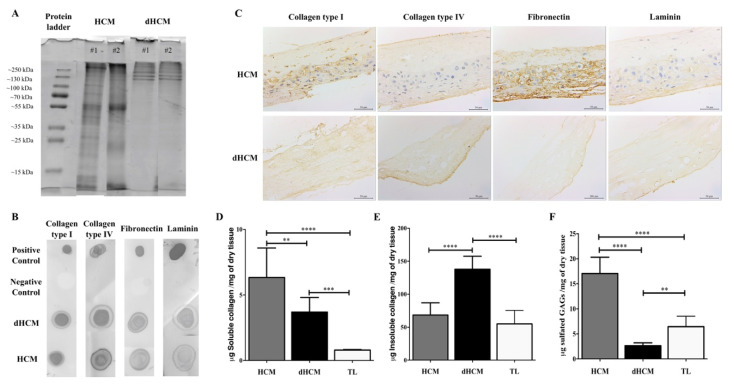
dHCM protein characterization. Representative lanes of two different samples from an SDS-PAGE gel of the digested HCM and dHCM (**A**). Representative dot blot results for collagen type I, collagen type IV, fibronectin and laminin, in both native and dHCM tissues. Digestion buffer without samples was used as a negative control, while the native protein was used as a positive control (**B**). Immunolocalization of collagen type I, collagen type IV, fibronectin and laminin in representative transversal sections of HCM and dHCM (**C**). Quantification of soluble collagen (**D**), insoluble collagen (**E**) and sulfated glycosaminoglycans (GAGs) (**F**) content in HCM, dHCM and the trophoblast layer (TL) in μg/mg of dry tissue. ** *p* ≤ 0.01; *** *p* ≤ 0.001 and **** *p* ≤ 0.0001.

The dHCM composition was assessed using SDS-PAGE, dot blot and immunolocalization of proteins. SDS-PAGE gel showed similarity among dHCM samples (Figure 2A), suggesting that the decellularization protocol is reproducible. Moreover, when compared to HCM, dHCM seems to preserve the higher molecular weight species (Figure 2A). By dot blot, it was possible to confirm the presence of some ECM high molecular weight proteins, such as collagen type I, collagen type IV, fibronectin and laminin, in both native and decellularized tissues (Figure 2B). To assess the distribution of these proteins, tissue sections of HCM and dHCM were used. As observed in Figure 2C, while collagen type I was spread along the dHCM, collagen type IV, fibronectin and laminin were focused on dHCM’s trophoblast side. Quantitative analysis was performed to detect the presence of soluble and insoluble collagen and sulfated glycosaminoglycans (GAGs) content in HCM, dHCM and trophoblast layer (TL). Regarding soluble collagen (Figure 2D), significant differences were observed between the HCM, dHCM and TL (*p* = 0.0045 (HCM vs. dHCM); *p* < 0.0001 (HCM vs. TL); *p* = 0.0006 (dHCM vs. TL)). Soluble collagen content was 6.33 ± 0.92 μg/mg of dry tissue in HCM, 3.70 ± 0.46 μg/mg of dry tissue in dHCM and 0.80 ± 0.06 μg/mg of dry tissue in TL. This tendency was not observed in insoluble collagen content (Figure 2E) where dHCM was associated with a higher quantity of insoluble collagen (137.90 ± 19.68 μg/mg of dry tissue) when compared with HCM (68.51 ± 18.51 μg/mg of dry tissue, *p* < 0.0001) and TL (55.09 ± 20.42 μg/mg of dry tissue, *p* < 0.0001). No statistically significant differences were observed between HCM and TL (*p* = 0.2850). The total collagen (soluble and insoluble) amount was significantly higher in dHCM (142.30 ± 20.07 μg/mg of dry tissue) when compared with HCM (74.78 ± 17.60 μg/mg of dry tissue, *p* < 0.0001) and TL (54.66 ± 20.92 μg/mg of dry tissue, *p* < 0.0001). No differences were observed between HCM and TL (*p* = 0.1406). For sulfated GAG quantification (Figure 2F), dHCM presented the lowest content (2.64 ± 0.19 μg/mg of dry tissue) when compared with HCM (17.07 ± 1.08 μg/mg of dry tissue, *p* < 0.0001) and TL (6.45 ± 2.09 μg/mg of dry tissue, *p* = 0.0040). Sulfated GAGs content in HCM was also significantly higher than in TL (*p* < 0.0001).

### 3.2. dHCM’s Mechanical Properties

**Figure 3:**
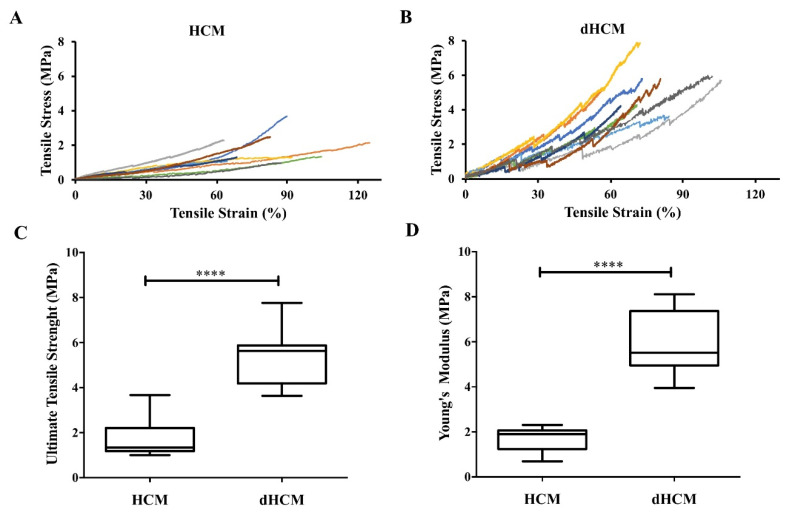
Mechanical properties of native and decellularized HCM. Stress-strain curves of HCM (**A**) and dHCM (**B**). Ultimate tensile strength (**C**) and Young’s modulus (**D**) of both HCM and dHCM. **** *p* ≤ 0.0001.

Mechanical properties were assessed in hydrated samples. Nine stress–strain curves are represented in Figure 3A,B for both native and decellularized tissue, respectively. At low strain levels, the stress varied linearly with the strain in accordance with Hooke’s law. At higher strain levels, membranes show a strain hardening behavior. The average ultimate tensile strength was 1.80 ± 0.28 MPa for native tissue and 5.33 ± 0.41 MPa for dHCM (Figure 3C). The average Young’s modulus was 1.69 ± 0.19 MPa for the native and 5.94 ± 0.47 MPa for the decellularized tissue (Figure 3D). Both ultimate tensile strength and Young’s modulus were significantly higher in dHCM (*p* < 0.0001).

### 3.3. Biocompatibility of dHCM In Vitro and In Vivo

**Figure 4:**
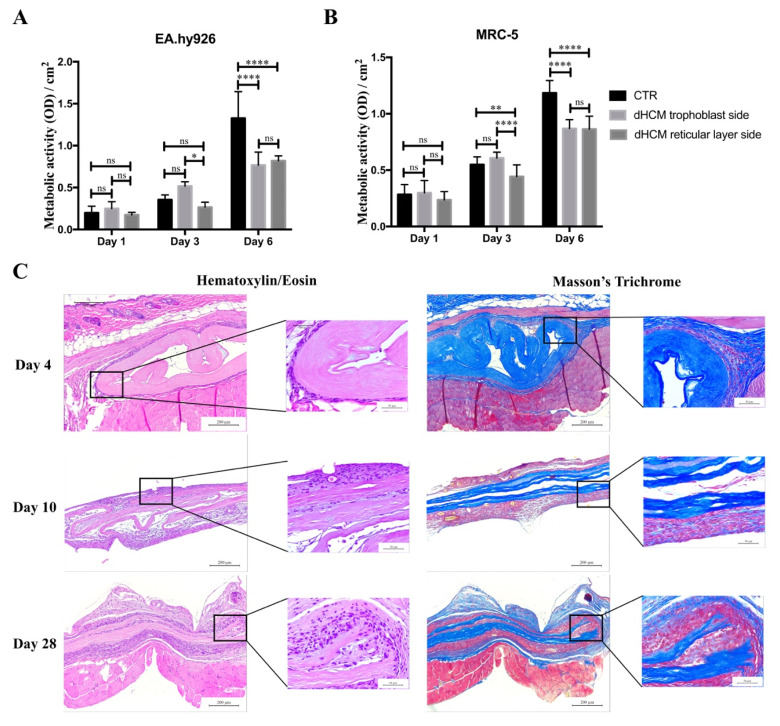
dHCM biocompatibility. EA.hy926 (**A**) and MRC-5 (**B**) cells’ metabolic activity in commercial inserts (CTR), dHCM trophoblast side and dHCM reticular layer side. Representative images of H&E and Masson’s trichrome staining of dHCM subcutaneously implanted on BALB/c mice at three different time-points (4, 10 and 28 days) (**C**). * *p* ≤ 0.05; ** *p* ≤ 0.001; and **** *p* ≤ 0.0001.

To evaluate the biocompatibility of dHCM in vitro, metabolic activity of EA.hy926 and MRC-5 cell lines were analyzed. Commercial inserts were used as controls (CTR). In all conditions, the metabolic activity of both EA.hy926 (Figure 4A) and MRC-5 (Figure 4B) cell lines increased over time. In general, both cell lines behaved in the same way. Regarding day 1, no differences were observed between conditions with the EA.hy926 cell line (*p* > 0.05) and with the MRC-5 cell line (*p* > 0.05). On day 3, the dHCM reticular layer side presented a significantly lower metabolic activity in both the EA.hy926 (*p* > 0.05 vs. dHCM trophoblast side) and the MRC-5 cell line (*p* < 0.05). Nevertheless, no differences were observed between CTR and both dHCM sides in the EA.hy926 cell line (*p* > 0.05) and between the CTR and dHCM trophoblast side in the MRC-5 cell line (*p* = 0.1365). Regarding day 6, CTR condition outperformed both dHCM sides with the EA.hy926 (*p* < 0.05) and the MRC-5 cell line (*p* < 0.05). No differences were observed between dHCM sides with both cell lines (*p* > 0.05). To further evaluate the biocompatibility of dHCM in vivo, dHCM membranes were subcutaneously implanted in BALB/c mice. After four, 10 and 28 days, animals were sacrificed and the tissue surrounding the dHCM membranes was collected for histological characterization. All animals survived during the period of the in vivo experiments. These animals did not demonstrate any mortality and infection throughout the study period, and the implantation of dHCM membranes did not produce any surgery-related and implantation-related abnormalities. As observed on Figure 4C, H&E staining demonstrated that on day 4, dHCM was surrounded by a thin layer of cells. Masson’s trichrome staining showed that the cell layer surrounding dHCM was also composed by collagen fibers (Figure 4C). In fact, this fibrotic capsule, composed by fibroblasts and collagen fibers, increased on day 10. Regarding day 28, an integration of native cells into dHCM was observed. Representative H&E staining from sham animals and from empty pockets (on the contralateral side of animals implanted with dHCM) are shown in Appendix A.

## 4. Discussion

In this study, a natural biomaterial was obtained resulting from a decellularization protocol previously developed by us for the HCM alone [27]. dHCM was characterized for its composition, mechanical properties and in vitro and in vivo biocompatibility.

In order to avoid cell and host adverse response, a decellularized tissue should (1) lack nuclear material (stained with DAPI); (2) have no DNA fragments larger than 200 bp; and (3) present an amount of dsDNA/mg of dry tissue less than 50 ng [14]. All these criteria were achieved by our process to obtain dHCM. During the decellularization process, the TL was removed and we obtained a compact and easy to manipulate membrane. A combination of decellularization methodologies comprising mechanical, chemical, detergent and enzymatic techniques was used in this work. Although the use of SDS and Triton-X in tissue decellularization is associated with the removal of cellular components and nuclear remnants, it is also associated with collagen damage, disruption and removal of GAGs [14]. This was observed in our results and it is in accordance with previous reports, since there was a decrease in the quantity of soluble collagen [31] and sulfated GAGs [31,32,33] in dHCM and TL (both submitted to the decellularization process) when compared to HCM. However, insoluble collagen content increased (per mg of dry tissue) in dHCM when compared with HCM. In fact, the total collagen amount also follows this trend. This phenomenon has been previously reported [33,34,35,36]. Due to the removal of GAGs and other cellular proteins during the decellularization process, the amount of the remaining collagen tends to increase per dry weight [33,36]. Overall, the obtained results suggest that our decellularization process was efficient in removing cells and DNA without substantial ECM damage. SDS-PAGE, dot blot and immunohistochemistry’s tissue sections demonstrating the preservation of ECM proteins such as collagen type I, collagen type IV, fibronectin and laminin, corroborate the previous results. Altogether, it is demonstrated that the decellularization protocol used herein is adequate, since the exposure time to detergents is relatively short and not too aggressive to induce major ECM disruption. The maintenance of ECM proteins in dHCM is of great importance since it maintains the specific cell microenvironment and can guide cell behavior [37], giving structural support to the tissue and functional input to modulate cell performance and function [38]. In fact, other ECM-based membranes, such as porcine small intestinal submucosa (SIS) [39] and decellularized HAM (dHAM) [40], are also composed by these proteins. However, dHAM also presents collagen type III and V that were not studied in this work.

Young’s modulus represents the stiffness or resistance to elastic deformation under load of a biomaterial, and the ultimate tensile strength characterizes the resistance of a material to fracture. Both Young’s modulus and the ultimate tensile strength are higher in dHCM when compared to HCM (*p* < 0.0001), as already demonstrated [35]. This may be explained because the ECM is composed of several polymerized proteins that aggregate through non-covalent bonds, creating a fibrous network. The cells bind to the ECM through weaker bonds, the focal adhesions [41]. So, if the decellularization process is not too aggressive to damage the ECM proteins, it is expected that the native tissue (with weaker bonds) will show lower stiffness. Also the weaker cell–ECM bonds act locally as stress concentration or local structural defects, amplifying locally the stress field and leading to lower resistance to fracture of the native tissue [42,43]. The Young’s modulus of dHCM and ultimate tensile strength are both higher than SIS [44] but lower than acellular amnion membrane [45]. Nevertheless, the ability to suture dHCM was also indirectly demonstrated during our decellularization process, used in our case to identify the two sides of the dHCM.

The swelling behavior is an important parameter in the characterization of the dHCM which is associated with the stability of the membrane in aqueous medium. Thus, to perform this study, culture medium and PBS were used to characterize the stability of dHCM during cell culture and storage, respectively (Figure 1L). It was observed that after 30 min, the swelling behavior of dHCM stabilized. Thus, before each cell seeding stage, both membranes (dHCM and CTR) were immersed in culture medium overnight to ensure stable and comparable wetting of the membranes.

The cytocompatibility of dHCM was investigated using two human cell lines of endothelial cells (EA.hy926) and fibroblasts (MRC-5). Commercial inserts were used as reference. SEM micrographs (Figure 1C,D) and immunohistochemistry tissue sections (Figure 2C) demonstrated that after the decellularization protocol, a membrane with two different surfaces (reticular layer side and trophoblast side) was obtained. Both sides of dHCM showed to be cytocompatible with the two human cell lines (Figure 4A,B). Additionally, no differences were observed between the dHCM trophoblast side and the dHCM reticular layer side with the exception of day 3, which suggests better performance of the dHCM trophoblast side with both cell lines. On day 6, CTR condition outperformed both sides of dHCM (Figure 4A,B). This could be associated with SDS-associated toxicity. Nevertheless, in vivo subcutaneous results (Figure 4C) refute this assumption. So, it is hypothesized that the different surface areas of commercial inserts (0.33 cm^2^) and dHCM inserts (0.95 cm^2^) might influence cells to behave and organize in different ways [46], resulting in a distinct metabolic activity pattern over time between conditions. Moreover, corroborating this hypothesis, it is demonstrated that cellular metabolic activity increases significantly from day 3 to day 6 in both dHCM sides with both cell lines.

ECM scaffolds, as dHCM, are envisioned to help and accelerate the remodeling of injured tissues in vivo [47]. By its subcutaneous implantation in animal models, it is possible to obtain information about the elicited inflammatory response towards the biomaterial implanted and about its integration with the host tissue [48,49]. In summary, a healthy host response after biomaterial implantation is characterized by an acute proinflammatory phase mainly orchestrated by neutrophils, monocytes and macrophages, followed by an anti-inflammatory phase mediated by the transition from M1 to M2 macrophages. This response may end with fibroblast recruitment and collagen deposition that leads to a fibrous capsule formation [50,51,52]. So, fibrous capsule formation surrounding the biomaterial is a common response after subcutaneous implantation [53,54,55]. Nevertheless, the formation of a fibrous capsule was observed four days after implantation, suggesting a rapid recovery of the proinflammatory phase associated with biomaterial implantation. This also demonstrates that dHCM final washes with PBS are sufficient and efficient since it is free of DNase and detergents (such as SDS) associated with adverse immune responses in vivo [56]. Importantly, on day 28, it is possible to observe that dHCM membranes are being integrated by the host tissue, revealing the beginning of a tissue regeneration stage. This is in contrast with SIS which is almost completely infiltrated by host cells after one week subcutaneously [55]. However, SIS has been associated with higher recurrent rates in hernia repairs [57,58,59] that may be associated with rapid degradation and integration. So, the longer time until integration by host tissue observed with dHCM might be a positive point, allowing a sustained tissue regeneration and eventually decreasing unwanted/adverse effects such as hernia recurrence. Moreover, in general, dHCM and dHAM have similar behavior in vivo [40]. Altogether, these results support the hypothesis that dHCM may be used as a biomaterial for promoting tissue regeneration in case of hernias, barrier defects, wounds, burns and/or skin defects. However, further dHCM barrier perfusion and wound healing assays should be performed.

## 5. Conclusions

In this work, the dHCM was fully characterized, for the first time, for the presence and distribution of ECM components, mechanical properties and in vivo and in vitro behavior. It was demonstrated that the dHCM is in accordance with the established criteria for decellularized tissues to avoid cell and host adverse reactions [14]. As SIS and dHAM, dHCM preserved some important ECM proteins such as collagen type I, collagen type IV, fibronectin and laminin and showed to be biocompatible in vitro and in vivo in subcutaneous implantation. Nevertheless, as a differentiator characteristic, dHCM has two different surfaces (reticular layer side and trophoblast side) that have different composition and topography. These results, together with the ability to suture dHCM and its mechanical properties, support the hypothesis of dHCM to be used as a biomaterial for tissue regeneration applications, particularly when a membrane is needed to separate tissues, organs or other biologic compartments.

## 6. Patents

PCT/IB2020/056426.

## Figures and Tables

**Figure A1:**
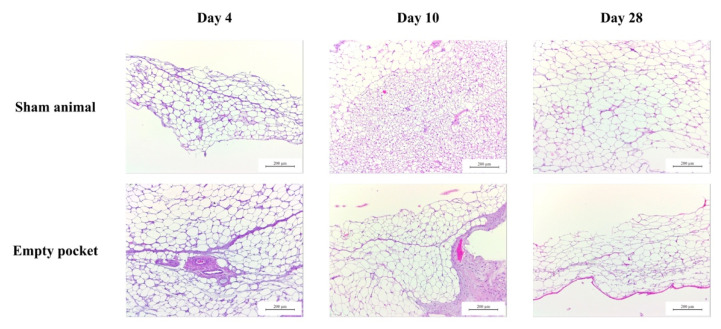
Representative images of H&E staining of control animals used in the subcutaneous assay performed on BALB/c mice at three different time-points (4, 10 and 28 days). In sham animals, only the surgery was performed (without dHCM implantation). Empty pockets were performed on the contralateral side of animals implanted with dHCM.
